# Supplementary material for: Computational Analysis of the Crystal and Cryo-EM Structures of P-Loop Channels with Drugs
Source: Int J Mol Sci. 2021 Jul 29;22(15):8143. doi: 10.3390/ijms22158143 (PMC8348670; doi:10.3390/ijms22158143)
Supplement: Supplementary file 1 [file ijms-22-08143-s001.zip › ijms-1312219-supplementary.pdf]

Supplementary Data

Computational Analysis of the Crystal and Cryo-EM Structures of P-loop Channels with Drugs

Denis B Tikhonov[1*] and Boris S Zhorov[1,2]

[1]Sechenov Institute of Evolutionary Physiology and Biochemistry, Russian Academy of Sciences, St. Petersburg; [2]McMaster University, Hamilton, Canada

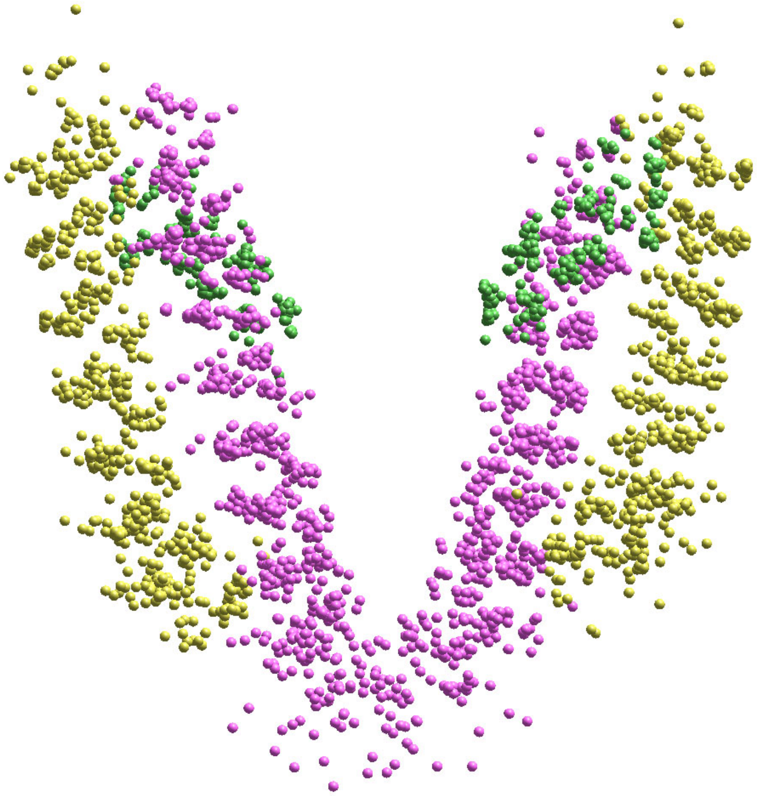

Figure S1. Alpha carbons in 3D-aligned experimental structures of ligand-bound P-loop channels. Yellow, green and magenta dots show, respectively CA atoms in the outer helices, pore helices and inner helices. PDB IDs: 2hvj, 2wof, 4xdk, 4xdl, 5klg, 5kls, 5kmh, 5un1, 6agf, 6dm0, 6jp5, 6jp8, 6jpa, 6jpb, 6juh, 6ke5, 6keb, 6kzo, 6kzp, 6mvx, 6uz0, 6yz0, 7jpl, 7jpl, 7jpx

Energetics of ligand-channel interactions

The following pages are generated by the ZMM program. Each ligand-channel complex was MC-minimized starting from the respective experimental 3D structure. CA atoms of the channels and heavy atoms of ligands were constrained to receptive positions in the experimental structures by pins (See Methods). Shown are the energy of ligand-channel (receptor) interactions (kcal/mol), its components, and energy partitioned by channel residues. Residues that contribute energy |E|< 0.3 kcal/mol are not shown. Note that in the AMBER force field a large part of the H-bonding energy is a part of respective non-bonded interactions. Labels of ions and ligands start from characters "I" and "L", respectively, followed by residue number. The latter shows the appearance order of the ion or ligand in respective PDB file.

```
CRYSTAL STRUCTURE OF KCSA-FAB-TBA COMPLEX (PDB ID: 2hvj)

Receptor interactions with ligand TBA

Ligand-receptor H-bonds            0.000
Ligand-receptor van der Waals    -19.257
Ligand-receptor electrostatic     -1.180
Ligand-receptor desolvation        7.458
Ligand-receptor total            -12.979

Interaction energy partitioned by receptor residues (main contributors)

Label   Name    Energy
 I___2    K      7.33
 3i15   ILE    -2.40
 4i15   ILE    -2.40
 2i15   ILE    -2.09
 1i15   ILE    -1.98
 4p49   THR    -1.23
 2p49   THR    -1.07
 1i18   PHE    -0.94
 3p49   THR    -0.82
 3i18   PHE    -0.77
 2p48   THR    -0.59
 2i18   PHE    -0.59
 1p49   THR     0.59
 2p47   ALA    -0.55
 4p48   THR    -0.51
 1p47   ALA    -0.48
 4i18   PHE    -0.48
 4i16   THR    -0.39
```

POTASSIUM CHANNEL KCSA-FAB COMPLEX WITH TETRAOCTYLAMMONIUM (PDB ID: 2wof)

Receptor interactions with ligand HX0

Ligand-receptor H-bonds	0.000
Ligand-receptor van der Waals	-34.928
Ligand-receptor electrostatic	-0.505
Ligand-receptor desolvation	14.179
Ligand-receptor total	-21.255

Interaction energy partitioned by receptor residues (main contributors)

Label	Name	Energy
I__65	K	6.51
1i15	ILE	-2.99
3I15	ILE	-2.62
3i18	PHE	-2.45
2i15	ILE	-2.15
4i15	ILE	-1.90
1i18	PHE	-1.73
4i18	PHE	-1.64
3p49	THR	-1.46
1p49	THR	-1.39
3p47	ALA	0.98
4p49	THR	-0.95
4p48	THR	-0.82
2i18	PHE	-0.80
2p49	THR	-0.71
2i14	GLY	-0.61
4i14	GLY	-0.60
2p47	ALA	-0.58
2p48	THR	-0.54
4p47	ALA	-0.53
4o13	LEU	-0.43
2o13	LEU	-0.41
2p50	VAL	-0.39
1o13	LEU	-0.39
3i17	SER	-0.38
2i11	MET	0.36
3o13	LEU	-0.35
4p50	VAL	-0.32

CRYSTAL STRUCTURE OF HUMAN TWO PORE DOMAIN POTASSIUM ION CHANNEL TREK2 (K2P10.1) IN COMPLEX WITH NORFLUOXETINE (PDB ID: 4xdk)

Receptor interactions with ligand L408

Ligand-receptor	H-bonds	-0.003
Ligand-receptor	van der Waals	-12.366
Ligand-receptor	electrostatic	1.862
Ligand-receptor	desolvation	8.298
Ligand-receptor	total	-2.208

Interaction energy partitioned by receptor residues (main contributors)

Label	Name	Energy
I___3	K	2.34
2i18	PHE	-1.69
2p47	LEU	-1.47
I___2	K	1.16
3i15	ILE	-1.09
3i12	ILE	-0.82
3i8	ILE	-0.72
L___5	L405	0.68
I___1	K	0.67
2i21	VAL	0.65
2p48	THR	-0.64
3i11	ALA	-0.55
2i27	ASP	-0.48

CRYSTAL STRUCTURE OF HUMAN TWO PORE DOMAIN POTASSIUM ION CHANNEL TREK2 (K2P10.1) IN COMPLEX WITH A BROMINATED FLUOXETINE DERIVATIVE (PDB ID: 4xdl)

Receptor interactions with ligand L40D

Ligand-receptor H-bonds	-0.000
Ligand-receptor van der Waals	-8.770
Ligand-receptor electrostatic	0.632
Ligand-receptor desolvation	3.527
Ligand-receptor total	-4.611

Interaction energy partitioned by receptor residues (main contributors)

Label	Name	Energy
3i12	ILE	-1.07
2p48	THR	-1.06
2i18	PHE	-0.87
2p47	LEU	-0.81
2i15	LEU	-0.50
2p44	VAL	-0.43
2p45	VAL	-0.36
2i14	GLY	0.35
3i11	ALA	-0.33
3i8	ILE	-0.30

STRUCTURE OF CAVAB(W195Y) IN COMPLEX WITH BR-DIHYDROPYRIDINE DERIVATIVE UK-59811 (PDB ID: 5klg)

Receptor interactions with ligand 6UC

Ligand-receptor H-bonds	-0.001
Ligand-receptor van der Waals	-13.634
Ligand-receptor electrostatic	-5.140
Ligand-receptor desolvation	4.936
Ligand-receptor total	-13.839

Interaction energy partitioned by receptor residues (main contributors)

Label	Name	Energy
2i22	VAL	-2.24
3i22	VAL	-1.52
3p47	MET	-1.15
3i16	PHE	-0.90
3p50	ASP	-0.81
2i18	MET	-0.78
I___2	CA	0.73
3p48	THR	-0.62
3i19	ILE	-0.48
3p49	LEU	-0.47
3I15	THR	-0.45
3p45	GLN	-0.41
I___1	CA	0.41
3i11	ILE	-0.39
4i18	MET	-0.36
2i26	ILE	-0.35
2p50	ASP	-0.34
4p49	LEU	-0.32

STRUCTURE OF CAVAB IN COMPLEX WITH BR-DIHYDROPYRIDINE DERIVATIVE UK-59811 (PDB ID: 5kls)

Receptor interactions with ligand 6UC

Ligand-receptor H-bonds	-0.045
Ligand-receptor van der Waals	-17.192
Ligand-receptor electrostatic	0.508
Ligand-receptor desolvation	9.079
Ligand-receptor total	-7.650

Interaction energy partitioned by receptor residues (main contributors)

Label	Name	Energy
3p40	PHE	-2.00
4i40	TRP	-1.92
4i8	ILE	-0.81
4i9	PRO	-0.56
3p41	TYR	-0.47
3p39	SER	-0.34
4p61	MET	-0.31
3p37	GLY	-0.26
3p44	PHE	-0.25

STRUCTURE OF CAVAB IN COMPLEX WITH BR-VERAPAMIL (PDB ID: 5kmh)

Receptor interactions with ligand 6U8

Ligand-receptor H-bonds	0.003
Ligand-receptor van der Waals	-19.160
Ligand-receptor electrostatic	2.635
Ligand-receptor desolvation	9.010
Ligand-receptor total	-7.513

Interaction energy partitioned by receptor residues (main contributors)

Label	Name	Energy
I___2	CA	11.18
3i18	MET	-2.39
3p49	LEU	-1.96
4p49	LEU	-1.55
2p49	LEU	-1.32
1i18	MET	-1.07
3p48	THR	-1.06
4i15	THR	-0.97
3p47	MET	-0.90
4p50	ASP	-0.86
I___1	CA	0.77
1p49	LEU	-0.71
1p47	MET	-0.63
2i15	THR	0.62
4i18	MET	-0.61
2p48	THR	-0.55
2i19	ILE	-0.48
4p48	THR	-0.47
1p50	ASP	-0.44
3p50	ASP	-0.40

CRYSTAL STRUCTURE OF GLUN1/GLUN2B DELTA-ATD NMDA RECEPTOR (PDBID: 5un1)

Receptor interactions with ligand BMK

Ligand-receptor H-bonds	-0.061
Ligand-receptor van der Waals	-15.679
Ligand-receptor electrostatic	-2.965
Ligand-receptor desolvation	8.226
Ligand-receptor total	-10.480

Interaction energy partitioned by receptor residues (main contributors)

Label	Name	Energy
3I15	MET	-1.39
2p49	ASN	-1.24
2i19	ALA	-0.86
1i17	VAL	-0.81
2i15	VAL	-0.75
2i22	THR	-0.64
4i18	LEU	-0.50
2i16	ILE	-0.50
4i22	THR	-0.48
1i18	ALA	-0.47
2p50	ASN	-0.38
1p48	LEU	-0.37
4i19	ALA	-0.34
4i15	VAL	-0.32
2i23	ALA	-0.30
2i18	LEU	-0.28

STRUCTURE OF THE HUMAN VOLTAGE-GATED SODIUM CHANNEL NAV1.4 IN COMPLEX WITH BETA1 (PDB ID: 6agf)

Receptor interactions with ligand 9Z9

Ligand-receptor H-bonds	-0.001
Ligand-receptor van der Waals	-27.898
Ligand-receptor electrostatic	-1.491
Ligand-receptor desolvation	4.280
Ligand-receptor total	-25.110

Interaction energy partitioned by receptor residues (main contributors)

Label	Name	Energy
3i27	ILE	-3.14
4i30	PHE	-2.53
1i26	ILE	-2.36
3i23	ILE	-2.03
4i22	TYR	-1.92
4i27	LEU	-1.83
L__10	6OU	-1.78
2i26	LEU	-1.45
4i23	ILE	-1.23
L__11	6OU	1.18
2i22	PHE	-1.02
2i30	PHE	-1.00
2i23	LEU	-1.00
1i23	LEU	-0.78
2i27	LEU	-0.59
1i27	ALA	-0.53
3i31	ASN	-0.52
3i19	LEU	-0.49
4i15	PHE	-0.49
4i28	GLU	-0.45

Receptor interactions with ligand 6OU (L10)

Ligand-receptor H-bonds	-0.337
Ligand-receptor van der Waals	-39.703
Ligand-receptor electrostatic	-31.056
Ligand-receptor desolvation	15.843
Ligand-receptor total	-55.253

Interaction energy partitioned by receptor residues (main contributors)

Label	Name	Energy
L__11	6OU	-13.08
3i16	PHE	-4.61
1p49	GLN	-2.92
3p48	THR	2.88
3p49	PHE	-2.75
4i15	PHE	-2.68
3i22	PHE	-2.44
4p48	THR	-2.32
1p48	THR	-2.17
3p47	ALA	-1.80
3i19	LEU	-1.71
3i12	ILE	-1.69
2p47	LEU	-1.67

3i11	ILE	-1.59
L__14	9Z9	-1.54
2i18	VAL	-1.37
2p49	GLY	-1.33
1p54	GLU	-1.21
3i17	PHE	-1.18
2p48	CYS	1.11
3i18	THR	-0.98
2i21	LEU	-0.97
2o13	LEU	-0.87
4p45	GLU	-0.77
3o17	TRP	-0.75
3I15	SER	-0.73
1i15	SER	-0.66
3o10	LEU	-0.64
3o13	CYS	-0.63
3o9	VAL	-0.61
2o10	THR	-0.60
3p50	LYS	-0.56
4p54	AS-	-0.54
2p44	PHE	-0.45
4i11	ILE	-0.44

Receptor interactions with ligand 6OU (L11)

Ligand-receptor H-bonds	-0.141
Ligand-receptor van der Waals	-28.683
Ligand-receptor electrostatic	-20.118
Ligand-receptor desolvation	18.391
Ligand-receptor total	-30.551

Interaction energy partitioned by receptor residues (main contributors)

Label	Name	Energy
L__10	6OU	-11.86
2i16	LEU	-2.57
1p44	PHE	-2.29
1o10	MET	-1.66
4o3	VAL	-1.47
L__14	9Z9	1.36
2i18	VAL	-1.27
2i12	VAL	-1.24
2i14	GLY	-1.22
1i22	ILE	-1.12
3i19	LEU	1.09
2p46	ILE	0.83
2i17	VAL	-0.79
1o6	LEU	-0.76
1i19	ILE	-0.74
2i15	ASN	-0.74
1p48	THR	-0.73
2i19	LEU	-0.65
2i21	LEU	0.63
1o17	LEU	-0.62
2p49	GLY	-0.60
2p47	LEU	-0.58
1p40	PHE	-0.57
3p47	ALA	-0.54

OPEN STATE GLUA2 IN COMPLEX WITH STZ AND BLOCKED BY IEM-1460, AFTER MICELLE SIGNAL SUBTRACTION (PDB ID: 6dm0)

Receptor interactions with ligand GZD

Ligand-receptor H-bonds	-0.091
Ligand-receptor van der Waals	-19.731
Ligand-receptor electrostatic	-12.128
Ligand-receptor desolvation	12.532
Ligand-receptor total	-19.419

Interaction energy partitioned by receptor residues (main contributors)

Label	Name	Energy
2p49	GLN	-3.65
4p49	GLN	-3.13
3p49	GLN	-2.07
3p50	GLN	-1.18
1p49	GLN	-1.13
1p50	GLN	-1.08
3p45	GLY	-0.93
4p52	CYS	-0.76
4p50	GLN	-0.75
3p53	ASP	-0.72
2p53	ASP	-0.67
4p53	ASP	-0.67
1p53	ASP	-0.56
2p52	CYS	-0.52
4i18	ILE	-0.45
2p51	GLY	-0.42
3p46	ALA	-0.41
4p45	GLY	-0.40
1p51	GLY	-0.39
1p46	ALA	-0.39

RABBIT CAV1.1-NIFEDIPINE COMPLEX (PDB ID: 6jp5)

Receptor interactions with ligand C5U

Ligand-receptor	H-bonds	-0.521
Ligand-receptor	van der Waals	-23.081
Ligand-receptor	electrostatic	-4.383
Ligand-receptor	desolvation	13.698
Ligand-receptor	total	-14.287

Interaction energy partitioned by receptor residues (main contributors)

Label	Name	Energy
3p44	PHE	-2.79
3i22	PHE	-2.13
3i19	MET	-1.84
3o13	THR	-1.18
4i11	TYR	-0.88
4i15	ALA	-0.73
3o17	GLN	-0.57
4i14	CYS	-0.54
3o6	ILE	-0.50
3p45	THR	-0.43
3o16	LEU	-0.38
3o10	VAL	-0.35
4i12	MET	-0.35
3i18	MET	-0.33
3p48	THR	-0.31
3I15	ALA	-0.26
3p47	SER	-0.25
3o9	ILE	-0.22
4i16	PHE	-0.21
3p49	PHE	-0.21

RABBIT CAV1.1-BAY K8644 COMPLEX (PDB ID: 6jp8)

Receptor interactions with ligand C8U

Ligand-receptor H-bonds	-0.698
Ligand-receptor van der Waals	-23.027
Ligand-receptor electrostatic	-3.633
Ligand-receptor desolvation	13.591
Ligand-receptor total	-13.767

Interaction energy partitioned by receptor residues (main contributors)

Label	Name	Energy
3p44	PHE	-3.20
3i19	MET	-2.29
3i22	PHE	-1.75
3p47	SER	-1.46
3o13	THR	-0.83
4i11	TYR	-0.71
3i18	MET	-0.57
3p45	THR	-0.57
3i14	ILE	-0.55
3o10	VAL	-0.51
3p48	THR	-0.43
4i16	PHE	-0.35
4i9	SER	-0.33
3o14	THR	-0.32
3p43	LEU	-0.29

RABBIT CAV1.1-VERAPAMIL COMPLEX (PDB ID: 6jpa mode 1)

Receptor interactions with ligand 4YH

Ligand-receptor H-bonds	-0.234
Ligand-receptor van der Waals	-28.895
Ligand-receptor electrostatic	-11.642
Ligand-receptor desolvation	14.972
Ligand-receptor total	-25.800

Interaction energy partitioned by receptor residues (main contributors)

Label	Name	Energy
L__11	3PE	-13.20
I___2	CA	9.56
L__12	9Z9	-2.90
2p47	LEU	-1.81
2i15	ASN	-1.74
3i22	PHE	-1.55
2i22	PHE	-1.42
3p49	PHE	-1.32
2p44	PHE	-1.17
2i19	LEU	-1.17
2p48	THR	-1.12
2p50	GL-	-1.06
2i18	LEU	-0.88
3p50	GLU	-0.86
1i23	LEU	-0.72
1i19	LEU	-0.71
I___1	CA	0.69
1p50	GLU	-0.56
3I15	ALA	-0.53
3i11	ILE	-0.45
2i11	PHE	-0.37
3i19	MET	-0.33
2o13	LEU	-0.30

RABBIT CAV1.1-VERAPAMIL COMPLEX (PDB ID: 6jpa mode 2)

Receptor interactions with ligand 4YH

Ligand-receptor H-bonds	-0.003
Ligand-receptor van der Waals	-31.042
Ligand-receptor electrostatic	-10.110
Ligand-receptor desolvation	13.669
Ligand-receptor total	-27.486

Interaction energy partitioned by receptor residues (main contributors)

Label	Name	Energy
L__11	3PE	-9.71
2i22	PHE	-3.35
1i16	PHE	-2.00
I___2	CA	1.67
L__12	9Z9	-1.48
4i18	ILE	-1.10
3p49	PHE	-1.02
2i18	LEU	-0.89
4i11	TYR	-0.89
4p47	ALA	-0.73
2i19	LEU	-0.59
I___1	CA	0.54
3i19	MET	-0.52
4i15	ALA	-0.49
3p50	GLU	-0.49
4p48	THR	-0.43
1p49	MET	-0.42
1i19	LEU	-0.40
3p48	THR	-0.35
3i22	PHE	-0.34

Receptor interactions with ligand 3PE

Ligand-receptor H-bonds	-0.069
Ligand-receptor van der Waals	-20.826
Ligand-receptor electrostatic	-101.689
Ligand-receptor desolvation	23.459
Ligand-receptor total	-99.125

Interaction energy partitioned by receptor residues (main contributors)

Label	Name	Energy
I___2	CA	-89.15
I___1	CA	-13.10
3p50	GLU	10.27
L__14	4YH	-9.68
2p50	GLU	8.80
1p50	GLU	7.89
2i12	VAL	-3.17
2i16	TYR	-2.94
4p50	GLU	2.48
2p47	LEU	1.97
1p47	ILE	-1.61

2i11	PHE	-1.55
2p46	VAL	-1.48
2p48	THR	1.27
1o-1	ILE	-1.27
3p51	GLY	1.17
1o13	VAL	-1.16
4p45	ARG	-1.05
4o3	MET	-1.04
3p47	SER	-1.03
4p54	GLU	0.99
1i19	LEU	-0.99
2p51	ASU	0.98
2i19	LEU	-0.95
4p47	ALA	-0.93
2p45	GLN	-0.91
1p54	ASP	0.90
2i15	ASN	-0.87
2p49	GLY	0.83
1p48	THR	-0.81
3p48	THR	0.80
3p52	TRP	-0.74
2i13	CYS	-0.72
4p48	THR	0.67
1p44	TYR	-0.67
4p49	GLY	0.63
2p52	TRP	0.49
2i17	ILE	-0.48
1i22	VAL	-0.48
1o6	LEU	-0.44

Receptor interactions with ligand 9Z9

Ligand-receptor H-bonds	-0.143
Ligand-receptor van der Waals	-26.731
Ligand-receptor electrostatic	-0.284
Ligand-receptor desolvation	7.077
Ligand-receptor total	-20.081

Interaction energy partitioned by receptor residues (main contributors)

Label	Name	Energy
4i22	PHE	-3.45
1i23	LEU	-1.73
3i26	VAL	-1.73
L__14	4YH	-1.60
1i26	ILE	-1.44
2i23	LEU	-1.36
4i23	VAL	-1.30
3i22	PHE	-1.08
4i19	ILE	-1.06
4i27	MET	-0.74
1i19	LEU	-0.73
3i19	MET	-0.69
2i26	ALA	-0.66
4i21	LEU	-0.62
3i23	VAL	-0.62
2i27	VAL	-0.55
2i30	LEU	-0.54

RABBIT CAV1.1-DILTIAZEM COMPLEX (PDBID: 6jpb)

Receptor interactions with ligand C9F

Ligand-receptor H-bonds	-0.000
Ligand-receptor van der Waals	-14.118
Ligand-receptor electrostatic	-3.685
Ligand-receptor desolvation	6.888
Ligand-receptor total	-10.915

Interaction energy partitioned by receptor residues (main contributors)

Label	Name	Energy
I___2	CA	2.84
3p49	PHE	-2.04
4i18	ILE	-1.33
3p50	GLU	-1.01
4i11	TYR	-0.94
4i15	ALA	-0.84
4p47	ALA	0.69
1i23	LEU	-0.56
2p50	GLU	-0.54
3p48	THR	-0.52
3i23	VAL	-0.52
I___1	CA	0.51
1i16	PHE	-0.48
4i22	PHE	-0.46
4i19	ILE	-0.45
3p47	SER	-0.43
1i15	SER	-0.39
2i19	LEU	-0.38
2p47	LEU	-0.37
1i20	ASN	-0.36
1p48	THR	-0.35
2i16	TYR	-0.33
4i20	ASN	-0.28
4i17	LEU	-0.28

STRUCTURE OF CAVAB IN COMPLEX WITH EFONIDIPINE (PSB ID: 6juh)

Receptor interactions with ligand C9X

Ligand-receptor H-bonds	-0.004
Ligand-receptor van der Waals	-23.644
Ligand-receptor electrostatic	0.769
Ligand-receptor desolvation	11.055
Ligand-receptor total	-11.824

Interaction energy partitioned by receptor residues (main contributors)

Label	Name	Energy
1p49	LEU	-2.01
2p49	LEU	-1.53
2i15	THR	1.19
I___1	CA	-1.18
4p49	LEU	-1.17
4i15	THR	-1.03
4i18	MET	-0.93
1i19	ILE	-0.92
1p47	MET	-0.86
3p49	LEU	-0.77
4p47	MET	-0.65
4i19	ILE	-0.62
3i18	MET	-0.60
1i15	THR	0.54
3p48	THR	-0.51
2p50	ASP	0.51
4p48	THR	-0.50
3p50	ASP	0.46
1p50	ASP	0.40
2i18	MET	-0.39
4i22	VAL	-0.34
1i18	MET	-0.31
2p51	ASP	0.29
2i16	PHE	-0.28

STRUCTURE OF CAVAB IN COMPLEX WITH DILTIAZEM AND AMLODIPINE (PDB ID: 6ke5)

Receptor interactions with ligand D6C

Ligand-receptor H-bonds	-0.002
Ligand-receptor van der Waals	-18.059
Ligand-receptor electrostatic	2.188
Ligand-receptor desolvation	9.493
Ligand-receptor total	-6.380

Interaction energy partitioned by receptor residues (main contributors)

Label	Name	Energy
I___2	CA	10.93
4p49	LEU	-1.95
I___3	CA	1.84
3p47	MET	-1.55
3p48	THR	-1.47
4p47	MET	-1.33
4p50	ASP	-1.15
3p49	LEU	-1.02
3i18	MET	-1.00
3I15	THR	-0.83
3p50	ASP	-0.80
1p50	ASP	-0.72
4p48	THR	-0.71
4i19	ILE	-0.68
3p44	PHE	-0.65
1p49	LEU	-0.64
I___1	CA	0.59
4i12	PHE	-0.53
2p48	THR	-0.49
4i11	ILE	-0.46
2p50	ASP	-0.41
4p45	GLN	-0.40

STRUCTURE BASIS FOR DILTIAZEM BLOCK OF A VOLTAGE-GATED CALCIUM CHANNEL (PDB ID: 6keb)

Receptor interactions with ligand D6C

Ligand-receptor H-bonds	-0.000
Ligand-receptor van der Waals	-15.035
Ligand-receptor electrostatic	-3.935
Ligand-receptor desolvation	5.924
Ligand-receptor total	-13.046

Interaction energy partitioned by receptor residues (main contributors)

Label	Name	Energy
4p49	LEU	-1.55
3p49	LEU	-1.20
3i18	MET	-1.15
3p47	MET	-1.09
I___2	CA	0.96
3I15	THR	-0.82
4i15	THR	-0.74
4p50	ASP	-0.74
3p48	THR	0.62
3p50	ASP	-0.55
4p47	MET	-0.52
2i18	MET	-0.51
2p48	THR	-0.51
3i19	ILE	-0.51
2p49	LEU	-0.50
1p49	LEU	-0.48
I___1	CA	0.44
4i18	MET	-0.44
1p50	ASP	-0.36
2p47	MET	-0.34
4i19	ILE	-0.33
1p48	THR	-0.24
4i16	PHE	-0.23

CRYO-EM STRUCTURES OF APO AND ANTAGONIST-BOUND HUMAN CAV3.1 (PDB ID: 6kzo)

Receptor interactions with ligand 3PE

Ligand-receptor H-bonds	-0.905
Ligand-receptor van der Waals	-24.815
Ligand-receptor electrostatic	-11.001
Ligand-receptor desolvation	22.972
Ligand-receptor total	-13.749

Interaction energy partitioned by receptor residues (main contributors)

Label	Name	Energy
I___2	CA	11.41
2p50	GLU	-4.69
I___1	CA	4.55
1p50	GLU	-3.71
3p49	LYS	3.14
3i12	LEU	-2.93
3p50	ASP	-2.65
2p49	GLN	-2.14
2i15	ASN	-1.89
2p46	ILE	-1.76
2i18	LEU	-1.67
2p48	THR	-1.48
2p47	LEU	1.09
2p51	ASP	-1.08
3i16	PHE	-1.07
2i21	LEU	-1.03
4p50	ASP	-0.99
2i11	MET	0.91
2o13	LEU	-0.88
2o14	MET	-0.76
2o10	CYS	-0.75
3I15	ALA	-0.73
2o6	VAL	-0.72
2p54	LYS	0.65
2i14	GLY	-0.65
3p54	ASP	-0.63
3i14	VAL	-0.55
4p48	THR	-0.53
4p45	ARG	0.48
2o17	ILE	-0.47
3p52	TRP	0.45
4i15	GLN	-0.43
2o3	MET	-0.40
1p54	ASP	-0.40

CRYO-EM STRUCTURES OF APO AND ANTAGONIST-BOUND HUMAN CAV3.1 (PDB ID: 6kzp)

Receptor interactions with ligand DRZ

Ligand-receptor H-bonds	-0.107
Ligand-receptor van der Waals	-27.715
Ligand-receptor electrostatic	-16.178
Ligand-receptor desolvation	14.927
Ligand-receptor total	-29.073

Interaction energy partitioned by receptor residues (main contributors)

Label	Name	Energy
L__11	3PE	-11.17
2p47	LEU	-3.21
2i15	ASN	-3.20
2i19	PHE	-3.11
3p49	LYS	2.60
I___2	CA	2.00
2p44	PHE	-1.66
3i22	PHE	-1.50
2p49	GLN	-1.29
1i20	ASN	-0.89
2o13	LEU	-0.67
2p48	THR	-0.67
1i23	LEU	-0.57
2i16	TYR	-0.51
3I15	ALA	-0.43
4i19	VAL	-0.42
2i20	ASN	-0.41
2i14	GLY	-0.40
2i18	LEU	-0.39
2p50	GLU	-0.33
3i18	VAL	-0.31
1p48	THR	-0.30
2o9	PHE	-0.28
3i11	LEU	-0.27
2i23	VAL	-0.27
4i15	GLN	-0.23

NAVAB VOLTAGE-GATED SODIUM CHANNEL, I217C, IN COMPLEX WITH CLASS 1C ANTI-ARRHYTHMIC FLECAINIDE (PDB ID: 6mvx)

Receptor interactions with ligand K4D

Ligand-receptor H-bonds	-0.022
Ligand-receptor van der Waals	-14.727
Ligand-receptor electrostatic	-11.383
Ligand-receptor desolvation	8.086
Ligand-receptor total	-18.046

Interaction energy partitioned by receptor residues (main contributors)

Label	Name	Energy
3p49	LEU	-1.82
1p49	LEU	-1.38
1p50	GLU	-1.29
2p50	GLU	-1.21
3p50	GLU	-1.15
2p49	LEU	-1.09
1i15	THR	-1.06
3p48	THR	-1.06
4p49	LEU	-1.00
1p47	MET	-0.89
4p50	GLU	-0.71
4p48	THR	-0.70
1p48	THR	-0.53
2p48	THR	-0.48
1i18	MET	-0.47
2i15	THR	-0.45
1i11	ILE	-0.39
2p45	GLN	-0.37
3p47	MET	-0.36
1p46	VAL	-0.32
3p46	VAL	-0.32

CARDIAC SODIUM CHANNEL WITH FLECAINIDE (PDB ID: 6uz0)

Receptor interactions with ligand K4D

Ligand-receptor H-bonds	-0.100
Ligand-receptor van der Waals	-19.324
Ligand-receptor electrostatic	-8.699
Ligand-receptor desolvation	10.033
Ligand-receptor total	-18.090

Interaction energy partitioned by receptor residues (main contributors)

Label	Name	Energy
3p50	LYS	2.80
3p49	PHE	-1.98
3i16	PHE	-1.87
2i22	PHE	-1.49
3p48	THR	-1.47
3p47	ALA	-1.40
2p50	GLU	-1.33
2p48	CYS	-1.10
4i15	PHE	-0.84
1p50	ASP	-0.72
2p47	LEU	-0.72
3i19	LEU	-0.68
3p46	VAL	-0.68
1p49	GLN	-0.64
2p44	PHE	-0.51
4p54	ASP	-0.46
2p49	GLY	-0.46
2p54	GLU	-0.38
2i18	VAL	-0.37
3i8	VAL	-0.36
4p50	ALA	-0.35

FULL LENGTH OPEN-FORM SODIUM CHANNEL NAVMS F208L IN COMPLEX WITH CANNABIDIOL (PDB ID: 6yz0)

Receptor interactions with ligand CD0

Ligand-receptor H-bonds	-0.206
Ligand-receptor van der Waals	-18.507
Ligand-receptor electrostatic	0.832
Ligand-receptor desolvation	9.192
Ligand-receptor total	-8.689

Interaction energy partitioned by receptor residues (main contributors)

Label	Name	Energy
4i18	VAL	-2.38
4i22	PHE	-2.22
L__12	CD0	1.49
4p49	LEU	-1.23
I___1	NA	1.00
4p44	PHE	-1.00
1i12	MET	-0.97
L___6	CD0	-0.72
3i22	PHE	-0.58
1i15	THR	-0.55
1p49	LEU	-0.47
4o13	LEU	-0.37
4i15	THR	-0.35
4p47	MET	-0.28
L___9	CD0	0.27
I___2	NA	0.21
4i20	ASN	-0.20
1p48	THR	-0.19

RABBIT CAV1.1 IN THE PRESENCE OF 10 MICROMOLAR (S)-(-)-BAY K8644 IN NANODISCS AT 3.4 ANGSTROM RESOLUTION (PDB ID: 7jpl)

Receptor interactions with ligand C8U

Ligand-receptor H-bonds	-0.075
Ligand-receptor van der Waals	-22.096
Ligand-receptor electrostatic	0.433
Ligand-receptor desolvation	9.612
Ligand-receptor total	-12.126

Interaction energy partitioned by receptor residues (main contributors)

Label	Name	Energy
3i22	PHE	-2.11
3p44	PHE	-2.09
4i12	MET	-1.35
3o10	VAL	-1.20
3i19	MET	-1.00
3o13	THR	-0.81
3i18	MET	-0.79
3p48	THR	-0.78
3o17	GLN	-0.64
4i11	TYR	-0.56
3p40	MET	-0.47
3o16	LEU	-0.45
3i20	ASN	-0.32
4i14	CYS	-0.31
3o9	ILE	-0.29
4i15	ALA	-0.27
3p47	SER	-0.24
3o14	THR	-0.23
4i13	LEU	-0.20
I___1	CA	-0.18
4i8	ILE	-0.18
3i14	ILE	-0.16
3i21	ILE	-0.14

RABBIT CAV1.1 IN THE PRESENCE OF 100 MICROMOLAR (R)-(+)-BAY K8644 IN NANODISCS AT 3.2 ANGSTROM RESOLUTION (PDB ID: 7jpw)

Receptor interactions with ligand VFY

Ligand-receptor H-bonds	-0.475
Ligand-receptor van der Waals	-21.469
Ligand-receptor electrostatic	-2.651
Ligand-receptor desolvation	12.556
Ligand-receptor total	-12.039

Interaction energy partitioned by receptor residues (main contributors)

Label	Name	Energy
3i22	PHE	-2.47
3p44	PHE	-1.62
3i19	MET	-1.43
4i12	MET	-1.29
3i14	ILE	-0.97
3p47	SER	-0.82
4i15	ALA	-0.67
3o13	THR	-0.50
4i16	PHE	-0.47
3i18	MET	-0.43
4i8	ILE	-0.39
3i10	TYR	-0.36
3p46	VAL	-0.31
4i14	CYS	-0.30
3o12	VAL	-0.25
4i13	LEU	-0.25
3o15	LEU	-0.24
3o11	LEU	-0.24
3p48	THR	-0.22
4i11	TYR	-0.18
3o10	VAL	-0.18
4i19	ILE	-0.16

RABBIT CAV1.1 IN THE PRESENCE OF 100 MICROMOLAR AMLODIPINE IN NANODISCS AT 2.9 ANGSTROM RESOLUTION (PDB ID: 7jpx)

Receptor interactions with ligand 6UB

Ligand-receptor H-bonds	-0.740
Ligand-receptor van der Waals	-30.246
Ligand-receptor electrostatic	-21.256
Ligand-receptor desolvation	24.308
Ligand-receptor total	-27.934

Interaction energy partitioned by receptor residues (main contributors)

Label	Name	Energy
L__11	POV	-11.66
3i22	PHE	-2.51
3p44	PHE	-2.36
I___1	CA	2.22
3p50	GLU	-1.59
3o13	THR	-1.49
2p50	GLU	-1.36
3p48	THR	0.87
4i12	MET	-0.81
1p50	GLU	-0.79
3p47	SER	-0.72
3p49	PHE	0.71
3o10	VAL	-0.61
4i15	ALA	-0.58
4i16	PHE	-0.56
3i10	TYR	-0.55
4p50	GLU	-0.51
4i11	TYR	-0.48
3i14	ILE	-0.40
3o16	LEU	-0.40
2p51	ASP	-0.37
3o9	ILE	-0.37
